# The Identification of Zinc-Finger Protein 433 as a Possible Prognostic Biomarker for Clear-Cell Renal Cell Carcinoma

**DOI:** 10.3390/biom11081193

**Published:** 2021-08-12

**Authors:** Simone O. Heyliger, Karam F. A. Soliman, Marilyn D. Saulsbury, Romonia Renee Reams

**Affiliations:** 1 Department of Pharmaceutical Sciences, School of Pharmacy, Hampton University, Hampton, VA 23668, USA; simone.heyliger@hamptonu.edu (S.O.H.); marilyn.saulsbury@hamptonu.edu (M.D.S.); 2 College of Pharmacy and Pharmaceutical Sciences, Florida A&M University, Tallahassee, FL 32307, USA; karam.soliman@famu.edu

**Keywords:** KRAB, zinc-finger, tumor microenvironment, BAP1, SETD2, clear cell renal carcinoma, immune response, transcription, ZNF433

## Abstract

Clear-cell renal cell carcinoma (ccRCC) is the most common and aggressive form of all urological cancers, with poor prognosis and high mortality. At late stages, ccRCC is known to be mainly resistant to chemotherapy and radiotherapy. Therefore, it is urgent and necessary to identify biomarkers that can facilitate the early detection of ccRCC in patients. In this study, the levels of transcripts of ccRCC from The Cancer Genome Atlas (TCGA) dataset were used to identify prognostic biomarkers in this disease. Analyzing the data obtained indicated that the KRAB-ZNF protein is significantly suppressed in clear-cell carcinomas. Furthermore, ZNF433 is differentially expressed in ccRCC in a stage- and histological-grade-specific manner. In addition, ZNF433 expression was correlated with metastasis, with greater node involvement associated with lower ZNF433 expression (*p* < 0.01) and with a more unsatisfactory overall survival outcome (HR, 0.45; 95% CI, 0.33–0.6; *p* = 8.5 × 10^−8^). Since ccRCC is characterized by mutations in proteins that alter epigenetic modifications and /or chromatin remodeling, we examined the expression of ZNF433 transcripts in ccRCC with wildtype and mutated forms of BAP1, KDMC5, MTOR, PBRM1, SETD2, and VHL. Analysis revealed that ZNF433 expression was significantly reduced in ccRCC with mutations in the BAP1, SETD2, and KDM5C genes (*p* < 0.05). In addition, the ZNF433 promoter region was highly methylated, and hypermethylation was significantly associated with mRNA suppression (*p* < 2.2 × 10^−16^). In silico analysis of potential ZNF target genes found that the largest group of target genes are involved in cellular metabolic processes, which incidentally are particularly impaired in ccRCC. It was concluded from this study that gene expression of ZNF433 is associated with cancer progression and poorer prognosis, and that ZNF433 behaves in a manner that suggests that it is a prognostic marker and a possible tumor-suppressor gene in clear-cell renal cell carcinoma.

## 1. Introduction

Renal cell carcinoma (RCC) is the most frequent malignancy in the kidneys, accounting for 75% of all kidney cancers and 2–3% of all cancers [1]. Clear-cell renal cell carcinoma (ccRCC) is the most common pathological subtype of RCC, originating from the proximal uriniferous tubules, and often presents an aggressive phenotype [2]. ccRCC is rarely detected early and is largely asymptomatic, and the clinicopathological risk factors cannot fully distinguish ccRCC patients [3]. At late stages, ccRCC has high morbidity and mortality and a poor prognosis, and is relatively insensitive to chemotherapy and radiotherapy [4]. Therefore, it is urgent and necessary to mine kidney-cancer datasets to identify potential biomarkers that can facilitate the early detection of ccRCC in patients.

Zinc-finger proteins act as transcription regulators, and some are differentially expressed in cancers [5]. These transcription factors are structurally diverse and have been shown to mediate numerous cellular processes, such as replication and repair, transcription and translation, metabolism and signaling, cell proliferation, and apoptosis [6]. KRAB-ZNF proteins makeup approximately one-third of zinc-finger proteins and are probably the largest group of transcriptional regulators in mammals [7,8]. Despite the ubiquitous nature of these proteins, relatively little is known about the function of the KRAB-ZNF individual members. Members of the KRAB-containing protein family contain at least one KRAB domain on the N-terminus and bind DNA through their C2H2 zinc-finger domains [8]. The zinc ion interacts with two cysteine and two histidine residues to create a stabilized folded structure that consists of two or three beta-strands and one alpha-helix [9,10]. The alpha-helix mediates DNA binding through non-covalent interactions within the DNA major groove; however, variations in the amino acid sequence of the finger domains and spacing, as well as in zinc-finger number and higher-order structure, may increase the ability to bind multiple different ligands such as RNA, DNA–RNA hybrids, and even proteins [5,9,10]. Nonetheless, KRAB-ZNF proteins are often tissue-specific, and are primarily thought to act as transcriptional repressors and/or epigenetic suppressors of gene expression [8,10,11,12].

Because of the sheer number of KRAB-ZNF proteins, their roles in oncogenesis have not been fully elucidated. ZNF471 was shown to be downregulated in esophageal squamous cell carcinoma (ESCC) cells, and restoration of expression leads to the induction of apoptosis and G0/G1 arrest, as well as inhibition of tumor cell colony formation [8]. Similarly, ZNF382 is downregulated in multiple cancer types, including leukemia and the solid tumors of the head and neck, lung, esophagus, colon, stomach, cervix, and breast, and serve as a tumor suppressor [8,13,14,15]. ZNF671 was found to be downregulated in brain, breast, lung, head, neck, and skin cancers, and overexpression inhibited the epithelial–mesenchyme transition (EMT), as well as cancer cell proliferation, migration, and invasion [16,17]. Taken collectively, KRAB-ZNF proteins may serve as tissue-specific tumor-suppressor genes.

Here, we report that the ZNF433, a KRAB zinc-finger transcription family member, is downregulated in clear-cell renal cell carcinoma, and its expression is reduced in a stage-specific manner. Moreover, lower expression is associated with higher tumor grades and poor overall survival.

## 2. Materials and Methods

### 2.1. Gene and Protein Expression Analysis

#### 2.1.1. UALCAN Analysis

In this study, UALCAN (http://ualcan.path.uab.edu/analysis.html (accessed on 5 May 2021)) was utilized to assess the levels of transcripts in clear-cell renal cell carcinomas from the TCGA dataset. UALCAN is an online, open-access platform that contains TCGA raw data, including gene expression, protein expression (CPTAC dataset), promoter methylation, miRNA expression, and clinicopathological data [18]. UALCAN allows users to query the gene of interest and compare its expression to clinicopathological features. Specifically, UALCAN was employed in our study to analyze the mRNA expression of ZNF433 in relation to the clinicopathological parameters (cancer stage, renal cancer subtypes, metastasis, and grade) of ccRCC.

#### 2.1.2. TNM Plotter

The TNM plotter (https://www.tnmplot.com (accessed on 8 May 2021)) is a web application that enables a real-time comparison of gene-expression changes between tumor, normal, and metastatic tissues amongst different types of platforms across all genes. The software was used to assess ZNF433 expression using the TCGA datasets—a direct comparison between tumor and normal tissues was made by running the Mann–Whitney test [19].

#### 2.1.3. TIMER2.0

TIMER2.0 was used to assess gene associations and co-expression patterns of genes across TCGA cancer types. It was also used to evaluate ZNF433 expression in ccRCC with wildtype and mutant forms of key transcriptional factors associated with ccRCC tumor initiation and progression. TIMER2.0 (http://timer.cistrome.org, accessed on 8 May 2021) provides a robust estimation of immune infiltration levels for The Cancer Genome Atlas (TCGA) or user-provided tumor profiles using six state-of-the-art algorithms [20,21]. In addition, TIMER2.0 provides modules for investigating the associations between immune infiltrates and genetic or clinical features, clinical outcomes, and exploring cancer-related associations in the TCGA cohorts [20,21]. All TCGA tumor data used in TIMER2.0, including transcriptome profiles, somatic mutation calls, somatic copy number variations, and patient clinical outcomes, were collected from the GDAC firehose website [20,21].

#### 2.1.4. Human Protein Atlas

The Human Protein Atlas (HPA) was used to ascertain whether the ZNF433 protein was differentially expressed in normal kidney and renal carcinomas (https://www.proteinatlas.org, accessed 14 May 2021) [22].

### 2.2. Overall Survival Analysis

The online Kaplan–Meier plotter program (https://kmplot.com/analysis, accessed on 7 May 2021) was used to determine the prognostic value of ZNF433 expression in clear-cell renal cell carcinoma. Specifically, Kaplan–Meier survival plots were generated to ascertain whether there was a correlation between ZNF433 expression and the overall survival (OS) of clear-cell renal cell carcinoma patients [23,24]. To assess the prognostic value of ZNF433, each percentile (of expression) between the lower and upper quartiles was computed, and the best-performing threshold was used as the final cutoff in a univariate Cox regression analysis. The Kaplan–Meier survival plot and the hazard ratio with 95% confidence intervals and logrank *p* value were calculated and plotted in R using the ‘‘survplot’’ function of the ‘‘survival’’ Bioconductor package [23,24].

### 2.3. Methylation Analysis

Methylation status was assessed using MethSurv (https://biit.cs.ut.ee/methsurv, accessed on 9 May 2021). This web-based tool enables researchers to perform survival analysis for a CpG located in or around the proximity of a query gene using the TGCA dataset [25]. To ascertain if methylation status was associated with gene expression, we utilized the Shiny Methylation Analysis Resource Tool (SMART) web-based program. The SMART application integrates multiomics and clinical data with DNA methylation. It provides key interactive and customized functions, including CpG visualization, pan-cancer methylation profile, differential methylation analysis, correlation analysis, and survival analysis [26].

### 2.4. Gene Ontology and Pathway Analysis

The gene ontology (GO) enrichment analysis results of the genes co-expressed with ZNF433 was visualized using LinkedOmics (http://www.linkedomics.org, accessed on 9 July 2021). The LinkedOmics database contains multiomics data and clinical data for 32 cancer types comprising 11,158 patients from TCGA [27]. The “LinkFinder” module was used to investigate differentially expressed genes within the TCGA KIRC cohort. The “LinkInterpreter” module was used to perform GSEA pathway enrichment analysis using the GO Biological Process and KEGG databases. Results were analyzed for significance using the Pearson’s correlation test, with a *p*-value and false discovery rate (FDR or *q*-value) set at 0.05.

To determine if there were genes that were putative targets of ZNF433 transcription factors, we queried the Molecular Signatures Database (MSigDB) (https://www.gsea-msigdb.org/gsea/msigdb, accessed on 9 May 2021), which is a collection of annotated genes that can be used with the Gene Set Enrichment analysis software. The predicted target genes of ZNF433 (Table 1) were then uploaded into the PANTHER (Protein ANalysis THrough Evolutionary Relationships) Classification System analysis web to identify gene/protein networks that may be over-represented within the gene set. The PANTHER website (http://pantherdb.org/about.jsp, accessed on 9 May 2021) provides tools for functional analysis of lists of genes or proteins. For example, gene lists can be analyzed graphically in terms of sortable functional classes and pie or bar charts; or analyzed statistically by overrepresentation or enrichment tests [28,29,30,31].

### 2.5. Statistical Analysis

Statistical analysis of tumor and normal-tissue gene expression was conducted using the TNM plotter web analysis tool. Comparison of the normal and tumor samples was performed using a Mann–Whitney U test. Statistical significance was set at *p* < 0.05. In addition, the Kaplan–Meier survival plots with the number at risk, hazard ratio (HR), 95% confidence intervals (CI), and log-rank *p*-values were obtained using the Kaplan-Meier plotter web analysis tool. The *p*-value was set at *p* < 0.05 to indicate a statistically significant difference in overall survival in the high- and low-expression groups. For analysis of clinicopathological features, a T-test was conducted to analyze differential gene expression of ZNF433 between genders. One-way ANOVA was used to analyze ZNF433 gene expression across clinicopathological features. ROC, as well as Cox univariate and multivariate proportional hazard model analyses were performed using MedCalc software. Statistical significance was set at *p* < 0.05.

## 3. Results

### 3.1. ZNF433 mRNA and Protein Expression in Normal and Clear-Cell Renal Cell Carcinoma

Table 1 provides the types of clinicopathological features, the corresponding numbers of each phenotype that were contained in the TCGA KIRC dataset, and the significance of clinicopathological features relevant to differential expression of ZNF433.

**Figure 1:**
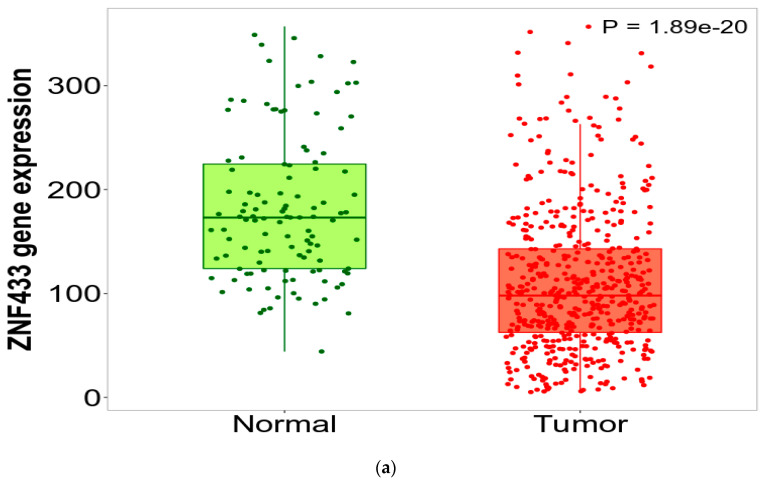

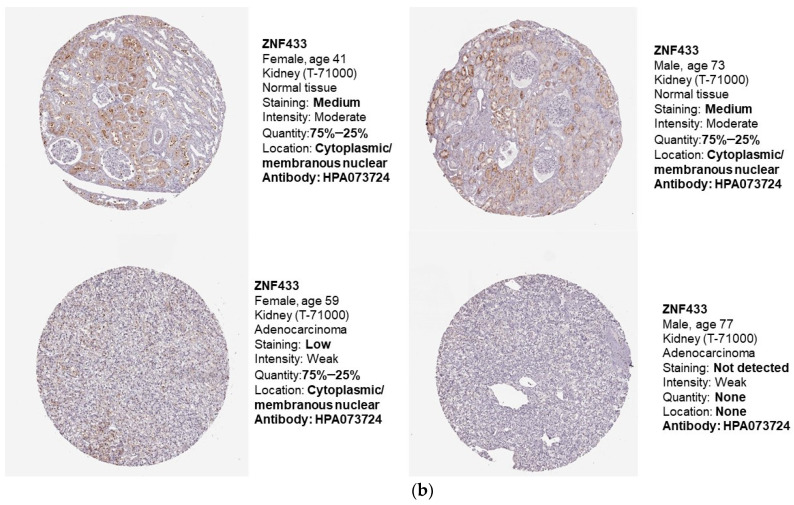
Expression of ZNF433 transcripts and protein in clear-cell renal cell carcinoma. (**a**) Plots were generated using TNMplot analysis (https://www.tnmplot.com, 14 May 2021) of RNAseq data using TCGA datasets. ZNF433 transcripts were significantly downregulated (fold change = 0.65) in tumors (*n* = 535) relative to normal tissues (*n* = 117). (**b**) Immunohistochemistry from the Human Protein Atlas website (https://www.proteinatlas.org, accessed on 14 May 2021) demonstrating expression of ZNF433 protein in normal and renal cancer tissues. The images are representative of normal kidney and renal adenocarcinoma tissues stained with anti-ZNF433 antibody (HPA073724). Representative images for tumors can be obtained at https://www.proteinatlas.org/ENSG00000197647-ZNF433/pathology/renal + cancer#img (accessed on 14 May 2021), and representative images for normal kidney tissue can be found at https://www.proteinatlas.org/ENSG00000197647-ZNF433/tissue/kidney (accessed on 14 May 2021).

Figure 1a depicts the expression of ZNF433 transcripts in the clear-cell renal cell carcinoma TCGA dataset. ZNF433 mRNA expression was significantly lower (*p* = 1.89 × 10^−20^) than in normal tissues. We examined Human Protein Atlas (https://www.proteinatlas.org/, accessed on 14 May 2021) immunohistochemistry images of human normal and renal carcinoma tissues stained with antibodies raised against the ZNF433 protein (Figure 1b) to validate the gene-expression data. The renal carcinoma images showed lower ZNF433 protein expression, as documented by the descriptions accompanying each image (https://www.proteinatlas.org/ENSG00000197647-ZNF433/pathology/renal+cancer#img, accessed on 14 May 2021). Renal cancer tissues had “low” or “not detected” staining with “weak” intensities, whereas all normal tissues were described as having “medium” staining with “moderate” intensities (Figure 1b), thereby suggesting that the ZNF433 protein is downregulated in renal carcinomas.

### 3.2. ZNF433 mRNA Expression in Association with the Clinicopathological Features of Clear-Cell Renal Cell Carcinoma

**Figure 2:**
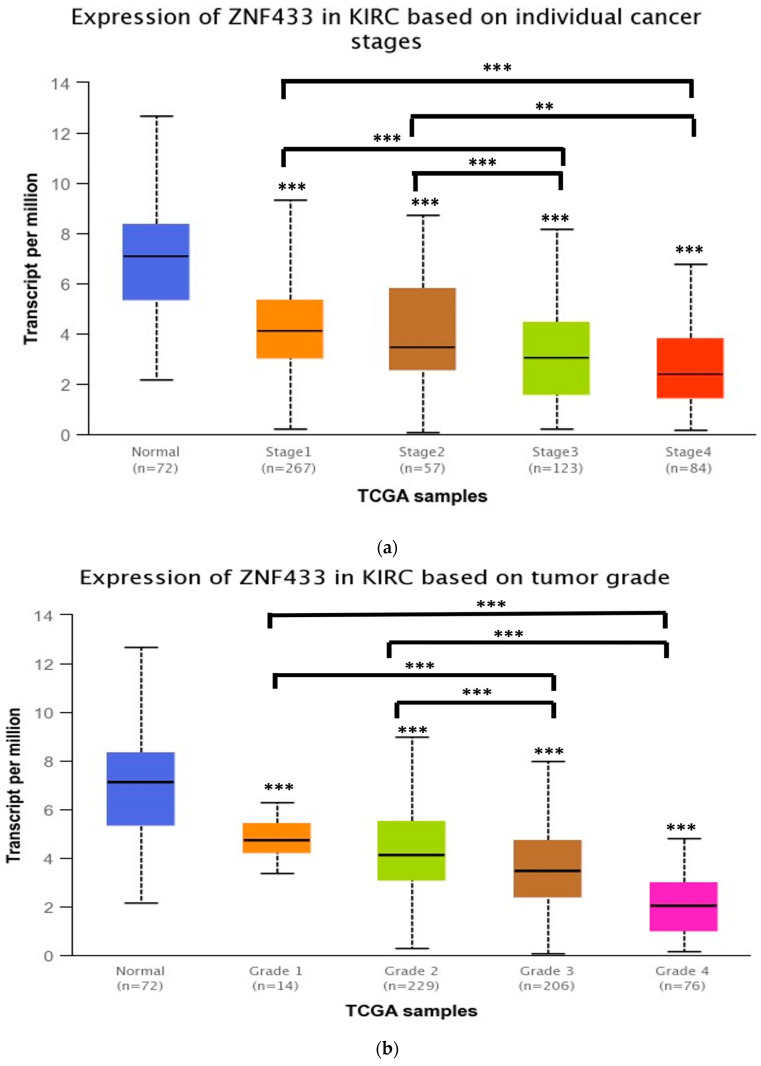

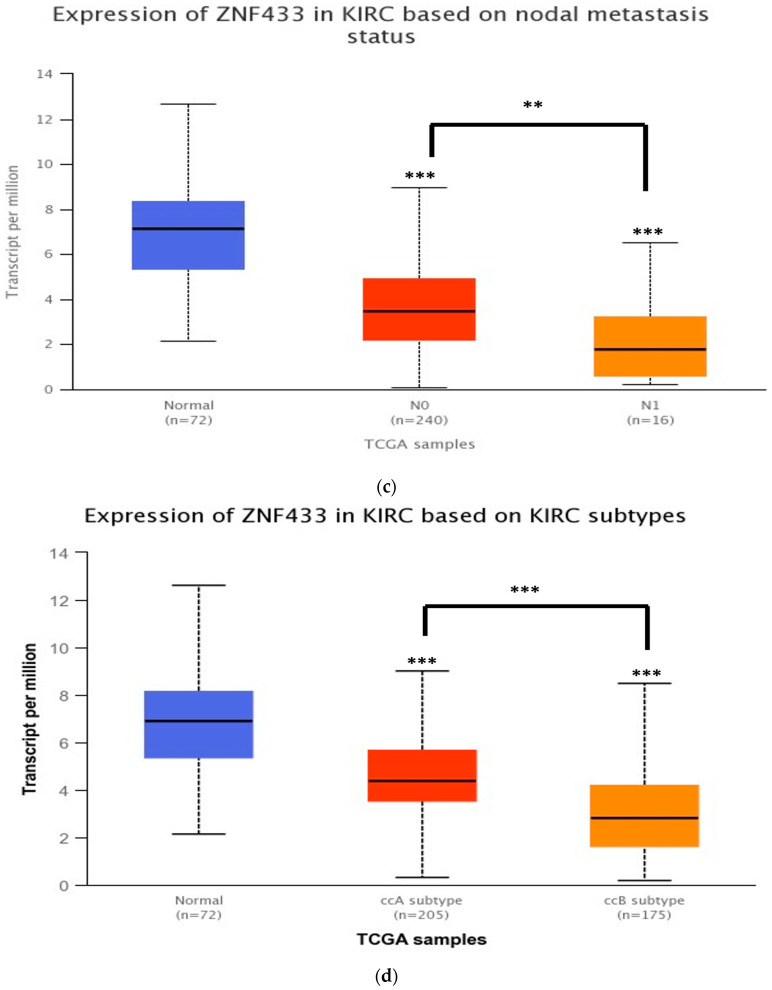
(**a**) Expression of ZNF433 transcripts across cancer stages in clear-cell renal cell carcinoma. ZNF433 transcripts were significantly downregulated in all tumor stages relative to normal tissues. (**b**) Expression of ZNF433 transcripts across histological grades of clear-cell renal carcinoma. ZNF433 transcripts were significantly downregulated in all tumor grades relative to normal tissues. (**c**) Expression of ZNF433 transcripts in metastatic tissues. ZNF433 transcripts were decreased with greater nodal involvement (*p* < 0.01). (**d**) Expression of ZNF433 transcripts in clear-cell renal cell carcinoma subtypes. ZNF433 transcripts were reduced in both subtypes, with ccB ccRCC exhibiting the lowest expression (*p* < 0.001). All plots were generated using UALCAN analysis of RNAseq data using TCGA datasets. ** *p* < 0.01 and *** *p* < 0.001.

To characterize ZNF433, we examined ZNF433 expression in different clinicopathological features of clear-cell renal cell carcinoma. ZNF433 remained suppressed (*p* < 0.001) across tumor stages (Figure 2a), with stages 3 and 4 having a lower expression of transcripts relative to normal as well as stages 1 and 2. A similar pattern was also noted regarding histological grades whereby ZNF433 expression was significantly downregulated in higher grades (3 and 4) (Figure 2b), (*p* < 0.01). In addition, significant downregulation of ZNF433 transcripts (Figure 2c) was noted across metastasis status, with ZNF433 expression decreasing significantly with more nodal involvement (*p* < 0.01) and thus higher metastatic status. Finally, ZNF433 expression was suppressed in both renal carcinoma subtypes (*p* < 0.001), with the more aggressive subtype ccB exhibiting the lowest level of mRNA expression (Figure 2d).

### 3.3. Overall Survival of Patients with Clear-Cell and Papillary Renal Cell Carcinomas as a Function of ZNF433 Expression

**Figure 3:**
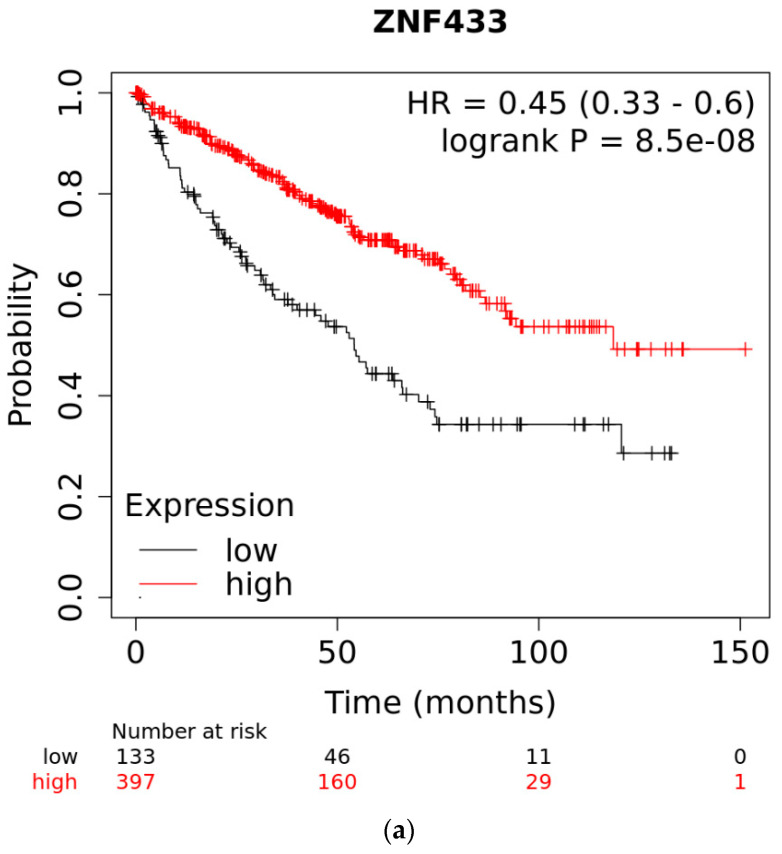

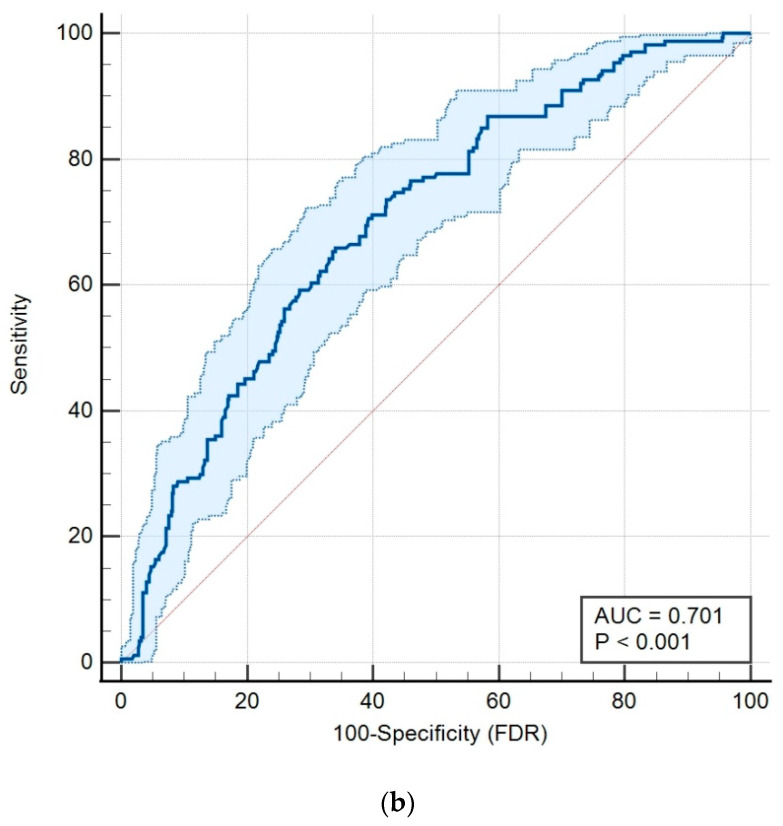
(**a**) Overall survival of patients with clear-cell and papillary renal cell carcinomas as a function of ZNF433 expression. The Kaplan–Meier survival plots were generated using K-M plotter analysis (https://kmplot.com/analysis, accessed on 7 May 2021) of RNAseq data from the clear-cell renal cell carcinoma (*n* = 533) TCGA datasets. The median survival for the high-expression group was 118.47 months, compared to 54.2 months for the low-expression group. (**b**) Receiver operating characteristic (ROC) curve of overall survival times of ZNF433 low- and high-expression patients. The ROC analysis was performed to validate KM survival observations and to ascertain if ZNF433 expression could discriminate between high-expression and low-expression groups in relation to survival. The AUC was 0.701, sensitivity = 0.66, specificity= 0.66, and 95% CI = 0.67–0.729 with *p* < 0.001.

A Kaplan–Meier survival analysis was performed to ascertain whether lower expression of ZNF433 is linked to changes in overall patient survival (Figure 3a). Lower expression of ZNF433 mRNA was associated with a poorer prognosis in patients with clear-cell renal cell carcinomas (HR, 0.45; 95% CI, 0.33–0.6; *p* = 8.5 × 10^−8^). The median survival for the high-expression group was 118.47 months, compared to 54.2 months for the low-expression group. A receiver operating characteristic (ROC) analysis was performed to validate KM survival observations and to ascertain if ZNF433 expression could discriminate between high-expression and low-expression groups in relation to survival. The AUC was 0.701, sensitivity = 0.66, specificity= 0.66, and 95% CI = 0.67–0.729 with *p* < 0.001. We performed Cox univariate and multivariate analyses on ZNF433 expression relative clinicopathological features (Table 2). In the univariate analysis, ZNF433 expression and histological grade, stage, and age were found to be predictive of overall survival. However, only age and stage retained significance in multivariate models. The ZNF433 *p*-value was 0.056, suggesting that there was an important tendency with regard to ZNF433 acting as a possible prognostic factor.

### 3.4. Effect of CpG Methylation on ZNF433 Gene Expression

**Figure 4:**
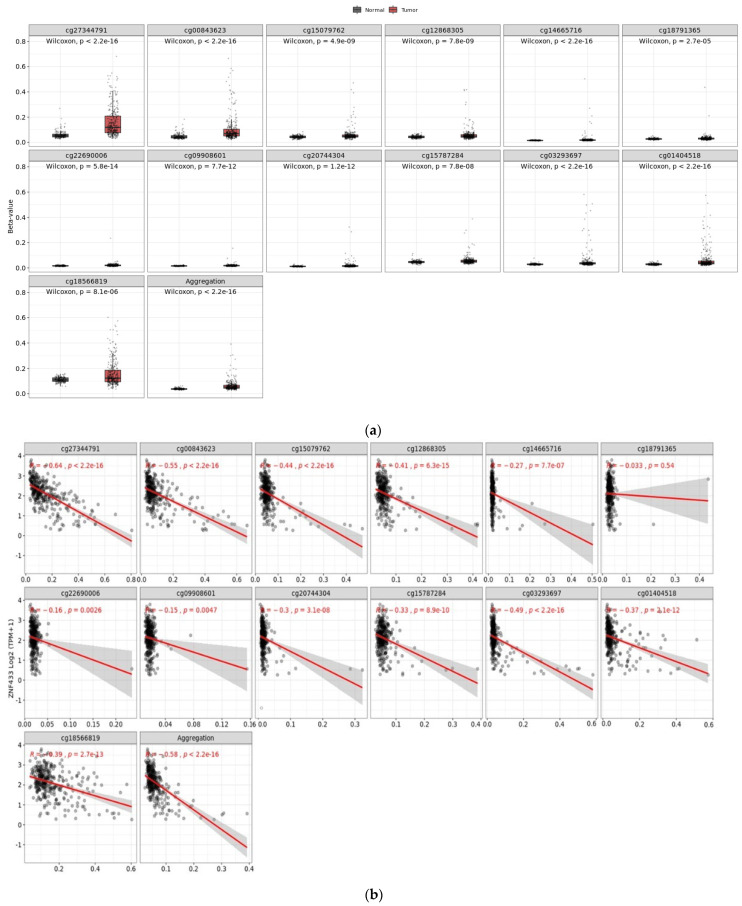

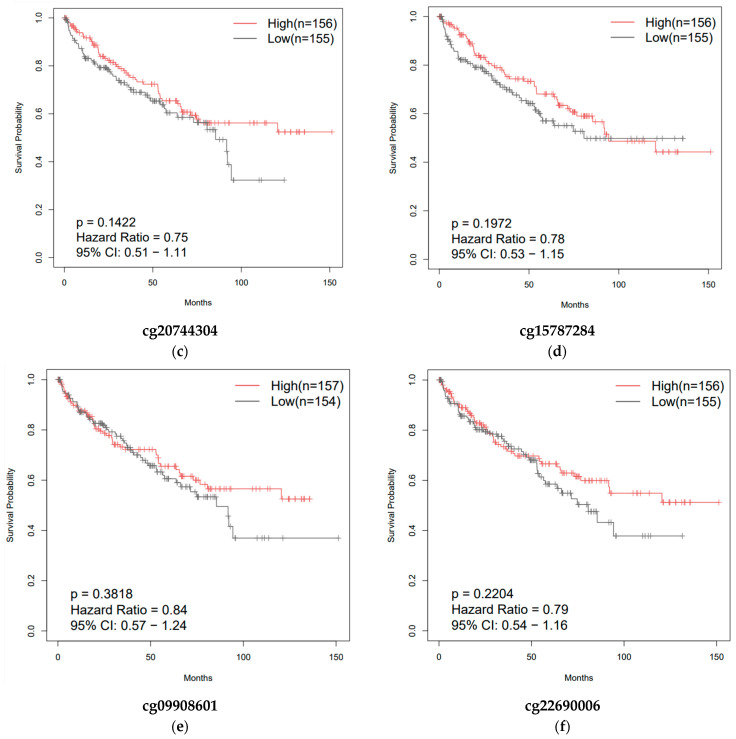

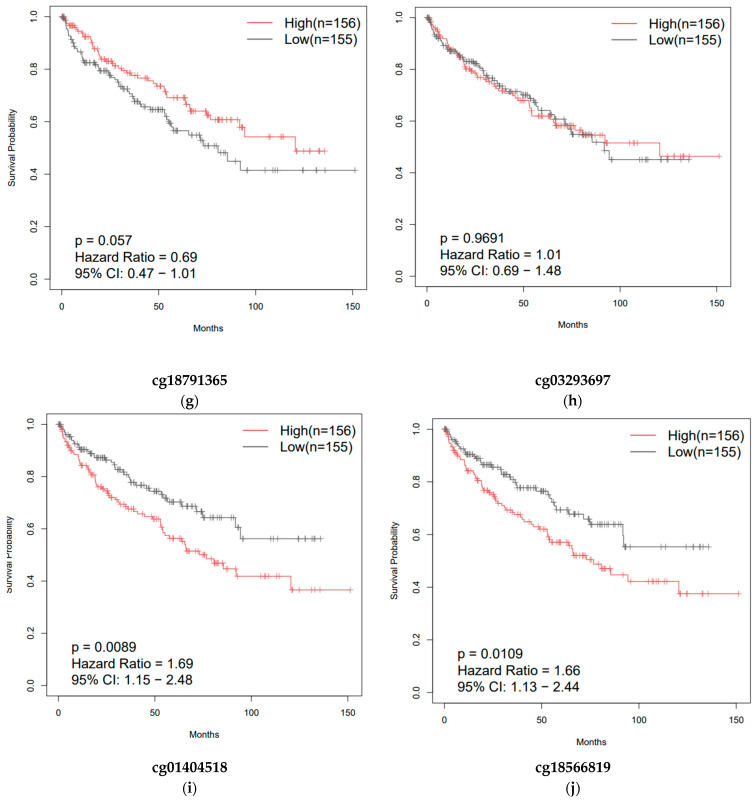
Methylation status of ZNF433 CpG loci and ZNF433 gene expression in normal and clear-cell renal cell carcinoma (ccRCC) tissues. (**a**) Methylation analysis between normal and ccRCC tissues revealed that CpG loci associated with ZNF433 were differentially methylated and statistically significant (*p* < 0.01). In addition, analysis of CpG foci in aggregate (last panel) revealed that the ZNF433 gene significantly hypermethylated (*p* = 2.2 × 10^−16^) relative to the normal tissues. (**b**) Correlational analysis between methylation status at CpG foci and ZNF433 protein expression revealed hypermethylation correlated with protein downregulation (*p* = 2.2 × 10^−16^). (**c**–**j**) KM survival plots for CpG islands that displayed differential methylation.

Given that zinc-finger transcription factors are highly regulated by methylation, we examined the methylation status of ZNF433 and compared it relative to mRNA expression (Figure 4a,b). The ZNF433 promoter region was highly methylated compared to the control (*p* = 1.62 × 10^−12^), and hypermethylation correlated with lower mRNA expression (R = −0.58; *p* < 2.2 × 10^−16^). Moreover, hypermethylation at promoter sites cg01404518 and cg18566819 sites was associated with significantly lower overall survival (*p* < 0.05) (Figure 4c–j).

### 3.5. Effect of BAP1, KDM5C, MTOR, PBRM1, SETD2, and VHL Mutations on ZNF433 mRNA Expression

**Figure 5:**
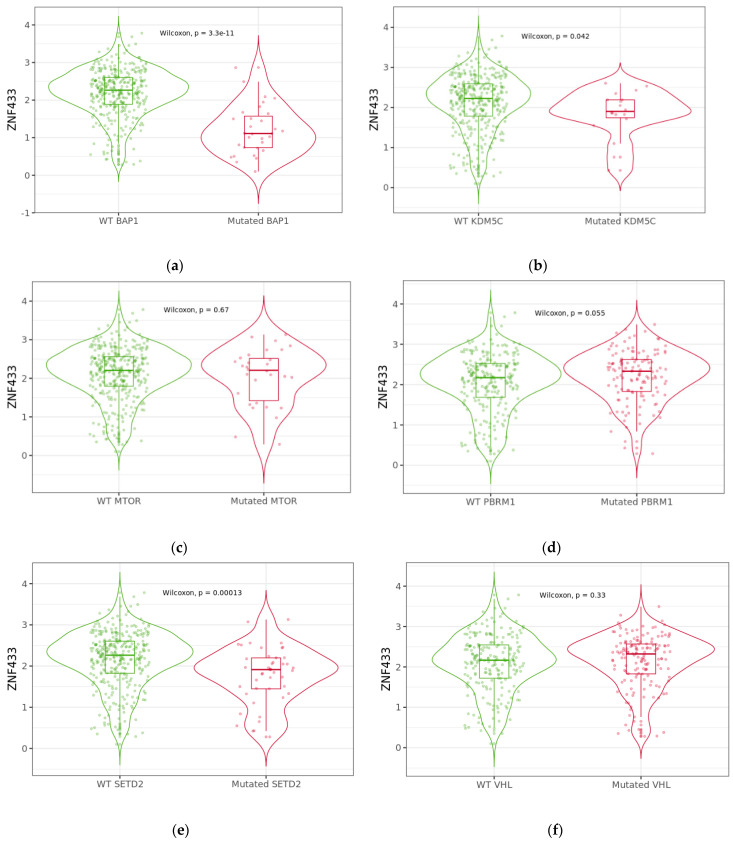
ZNF433 expression based on mutation status of key tumor-suppressor genes (figures **a**–**f**). Violin plots were generated using TIMER2.0 (http://timer.cistrome.org, accessed on 8 May 2021) to analyze ZNF433 expression between mutation status of the most frequently altered genes (**a**) BAP1, (**b**) KDM5C, (**c**) MTOR, (**d**) PBRM1, (**e**) SETD2, and (**f**) VHL that are associated with clear-cell renal cell carcinoma. ZNF433 expression was significantly altered in the presence of BAP1, KDM5C and SETD2. Expression analysis was conducted using RNAseq sample data (*n* = 367) derived from TCGA datasets. Abbreviations: BAP1, BRCA1 associated protein-1; KDMC5, lysine-specific demethylase 5C; mTOR, mechanistic target of rapamycin kinase; PBRM1, polybromo 1; SETD2, SET domain containing 2, histone lysine methyltransferase; VHL, von Hippel–Lindau tumor suppressor.

Since ccRCC is characterized by mutations in proteins that alter epigenetic modifications such as methylation and /or chromatin remodeling, we examined the expression of ccRCC with wildtype and mutated forms of BRCA1 associated protein-1 (BAP1); lysine-specific demethylase 5C (KDMC5); mechanistic target of rapamycin kinase (MTOR); polybromo 1 (PBRM1); SET domain containing 2, histone lysine methyltransferase (SETD2); and von Hippel–Lindau tumor suppressor (VHL) genes (Figure 5). ZNF433 expression was significantly depressed in ccRCC with mutations in BAP1 (*p* = 3.3 × 10^−11^), KDM5C (*p* = 0.042), or SETD2 (*p* = 0.00013) genes.

### 3.6. ZNF433 Pathway Enrichment, Target Gene Expression, and Target Gene Ontology

**Figure 6:**
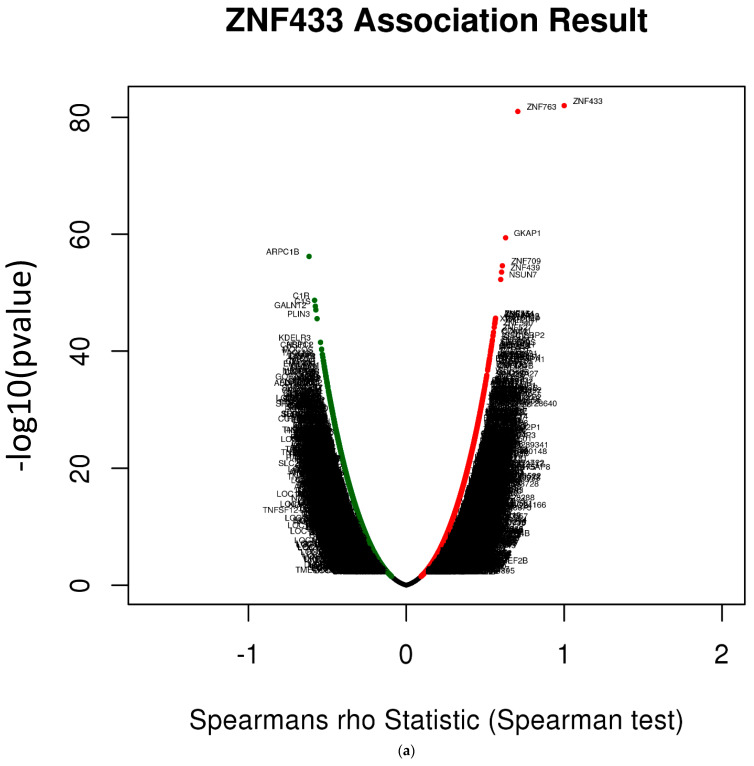

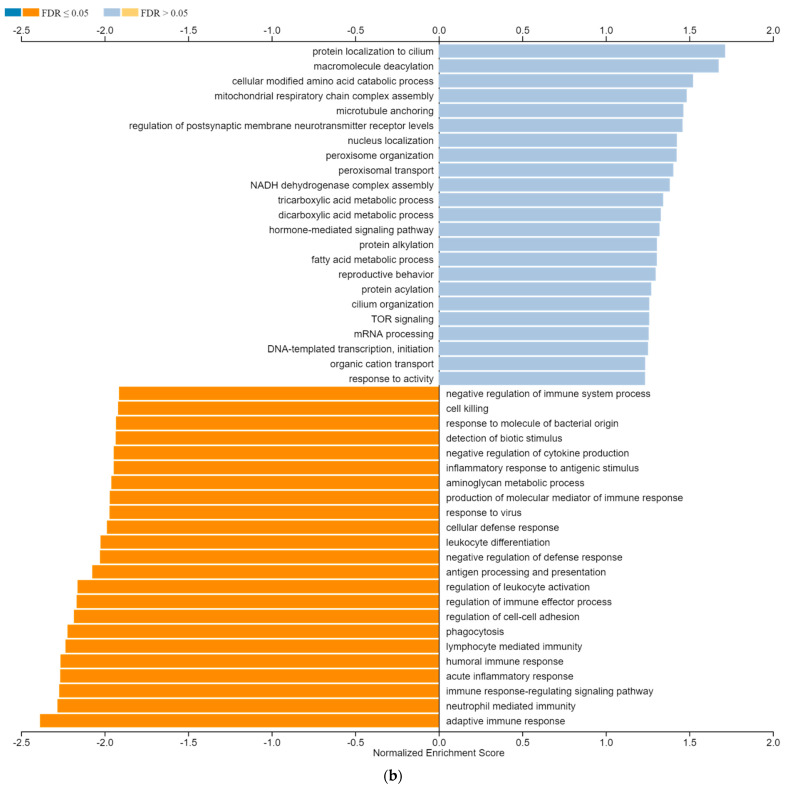

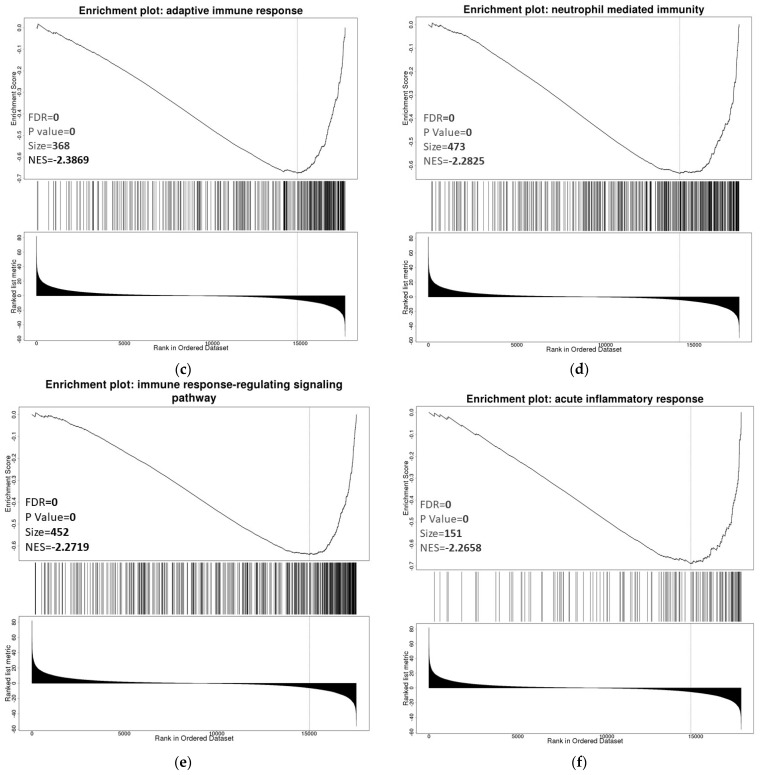

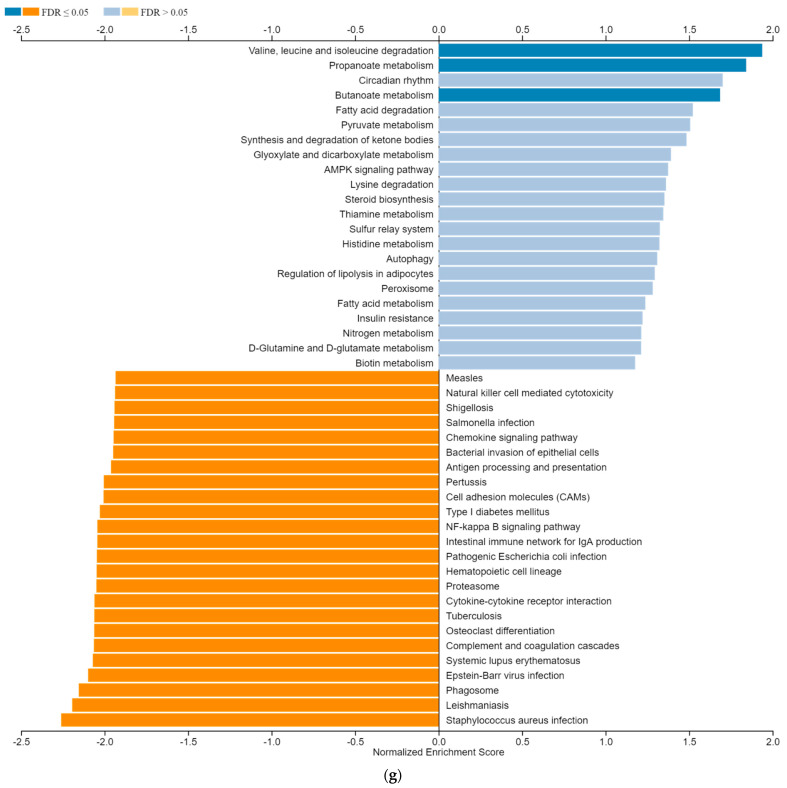

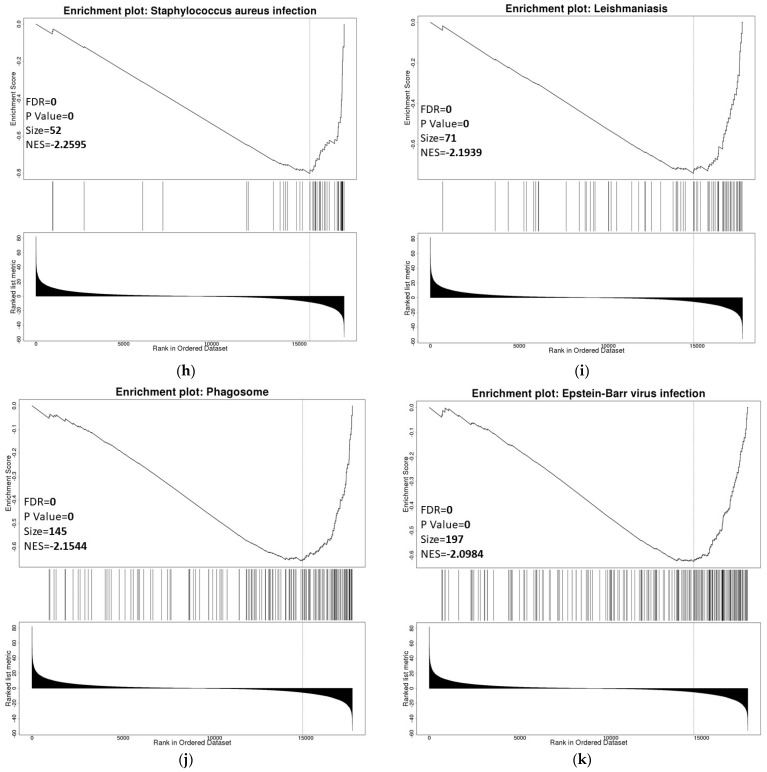
(**a**) Volcano plots of the negatively and positively correlated genes associated with ZNF433 expression. The green section of the volcano curve represents genes that were inversely correlated with ZNF433 expression, while the red region of the curve represents genes that were positively correlated with ZNF433. (**b**) GSEA GO Biological Process for genes co-expressed with ZNF433. Blue-colored bars represent pathways enriched in positively correlated genes, with dark blue bars representing FDR < 0.05, whereas light blue bars are pathways for which FDR > 0.05. Orange-colored bars represent pathways enriched in inversely correlated genes, with dark orange bars depicting pathways with FDR < 0.05, and light orange bars representing pathways with FDR > 0.05. (**c**–**f**) Enrichment plots for the top most-significant pathways that were over-represented among inversely correlated genes. (**g**) GSEA KEGG for genes co-expressed with ZNF433. (**h**–**k**) Enrichment plots for the top most-significant pathways over-represented among inversely correlated genes in the KEGG database.

Since there is limited information regarding ZNF433 target genes, we examined whether genes correlated highly with ZNF433 expression were components of over-represented pathways. Specifically, we used the gene set enrichment analysis (GSEA) method to analyze the GO Biological Process and KEGG databases to identify enriched pathways (Figure 6a–k). Figure 6a provides a volcano plot of the negatively (green line) and positively (red lines) correlated genes associated with ZNF433 expression. Figure 6b provides the GSEA analysis of the pathways enriched among genes positively (blue bars) and inversely (orange bars) co-expressed with ZNF433 using the GO Biological Process database. The only pathways that were significantly enriched (*p* < 0.05, FDR < 0.05) were those connected to the genes inversely co-expressed with ZNF433. Moreover, the enriched pathways were all associated with immune system response/regulation. Figure 6c–f provide the enrichment plots of the top four pathways enriched among the inversely co-expressed genes. These enriched pathways were the adaptive immune response (GO:0002250), neutrophil mediated immunity (GO:0002446), immune response-regulating signaling pathway (GO:0002764), and acute inflammatory response (GO:0002526). The most enriched pathways among the positively correlated genes included those associated with mitochondria/cellular metabolism and protein transport, including GO:0061512: protein localization to cilium (NES = 1.67, FDR = 0.2812, *p* = 0); GO:0098732: macromolecule diacylation (NES = 1.623, FDR = 0.262, *p* = 0.005); and GO:0033108: mitochondrial respiratory chain complex assembly (NES = 1.47, FDR = 0.55, *p* = 0.005). Similarly, GSEA analysis of the KEGG database revealed that immune-related pathways were predominantly enriched among inversely correlated genes (Figure 6g). Indeed, nine out of the 10 most significantly enriched pathways among inversely correlated genes were associated with defense against infectious organisms (Figure 6g). These included hsa05140 (Leishmaniasis), hsa04145 (Phagosome), hsa05150 (Staphylococcus aureus infection), and hsa05169 (Epstein–Barr infection). Their respective enrichment plots are provided in Figure 6h–k. For genes positively co-expressed with ZNF433, GSEA analysis of the KEGG database revealed that most significant pathways associated with cellular metabolism/energy were enriched, and included hsa00280: valine, leucine, and isoleucine degradation (NES = 1.93, FDR = 0.001, *p* = 0); hsa00640: propanoate metabolism (NES = 1.86, FDR = 0.005, *p* = 0); and hsa00650: butanoate metabolism (NES = 1.70, FDR = 0.04, *p* = 0).

**Figure 7:**
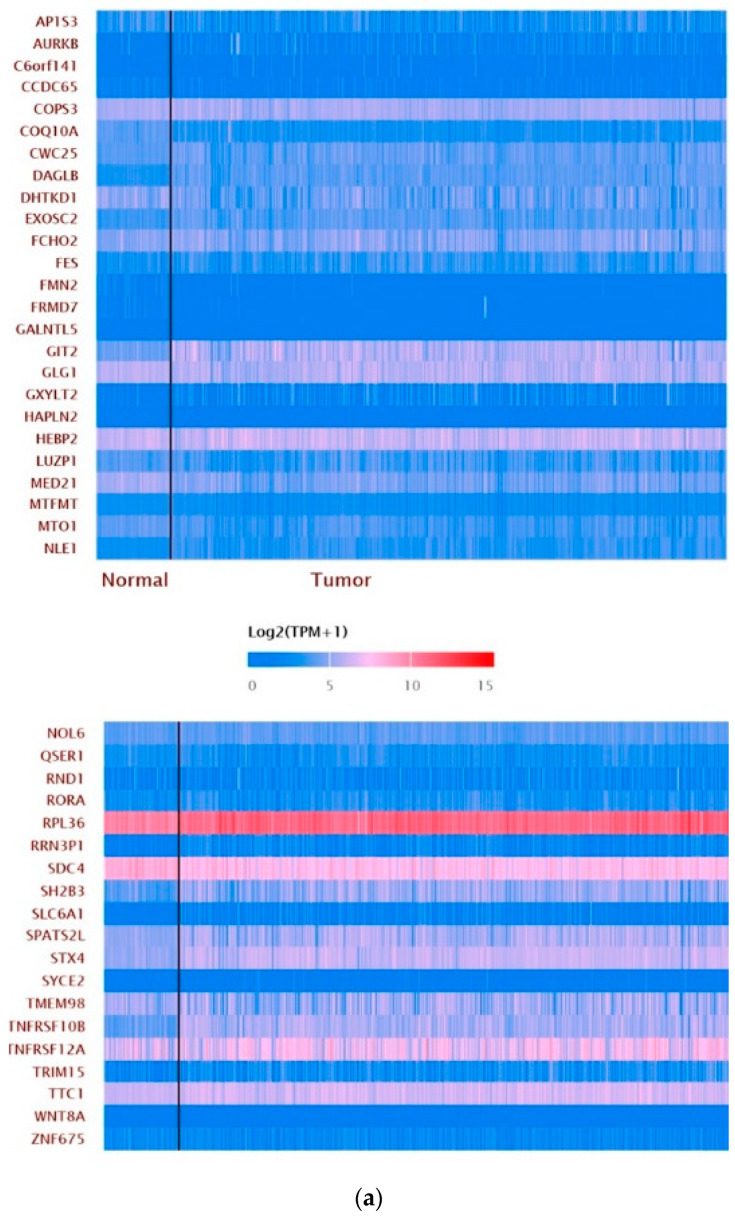

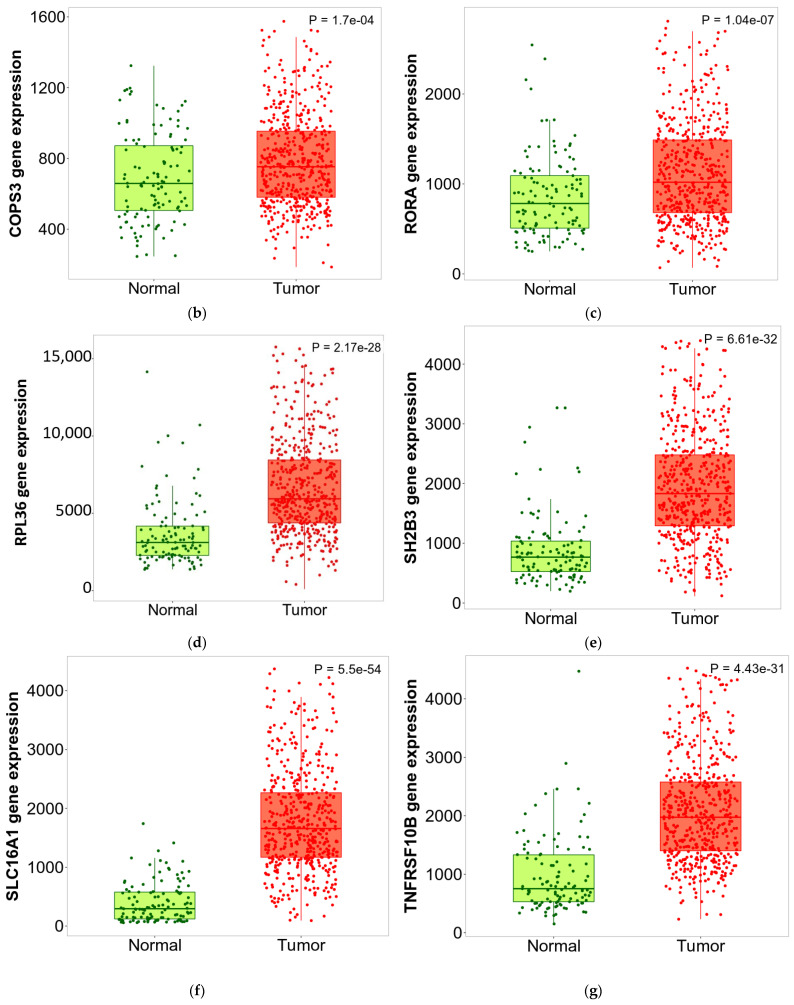

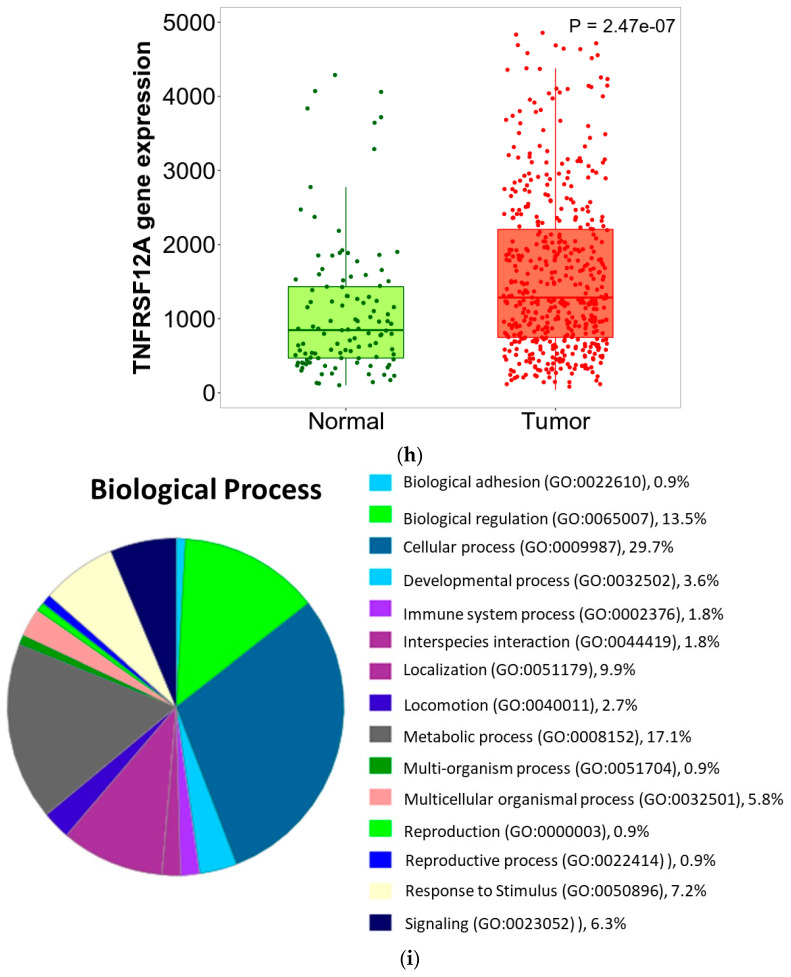

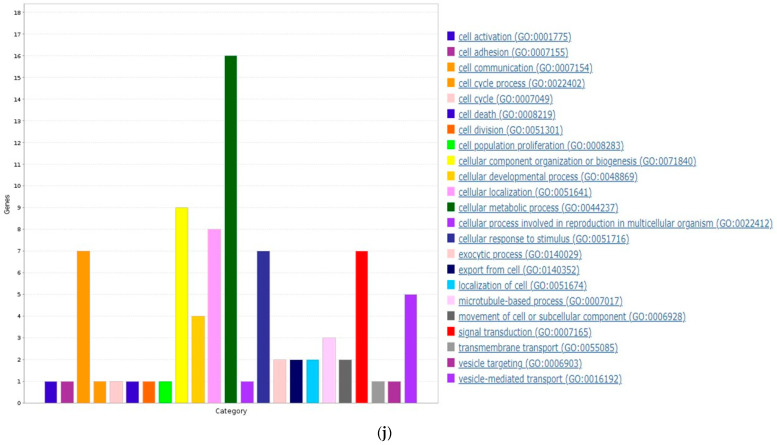
Expression of potential ZNF433 target genes in clear-cell renal carcinoma. (**a**) A heatmap generated using UALCAN analysis to determine the expression of predicted ZNF433 target genes in clear-cell renal cell carcinoma. (**b**–**h**) Most notable targets implicated in cancer suppression, progression, or tumorigenesis that had significant changes in ccRCC relative to normal, as well as those previously identified (*n* = 535). (**i**) A pie chart featuring biological processes in which target genes are components, generated using PANTHER (http:// pantherdb.org, accessed on 9 May 2021). (**j**) The largest category of the biological processes was a cellular process (GO: 0009987). The bar graph shows the number of ZNF433 target genes associated with the GO subcategories under the cellular process.

Since ZNF433 belongs to one of the most prominent transcription factor families, we utilized the GSEA Molecular Signature Database to identify 66 putative targets for ZNF433 (Table 2). These putative targets were genes that contained one or more binding sites for UniProt: Q8N7K0 (ZNF433) in their promoter regions (TSS −1000, +100 bp), as identified by GTRD version 20.06 ChIP-seq harmonization. Figure 7a depicts a heatmap generated from the ZNF433 target gene expression in clear-cell renal cell carcinoma and normal tissues. The most notable targets that showed significant changes in ccRCC relative to normal tissues, and that had been previously implicated in cancer suppression, progression, or tumorigenesis, were COPS3 (fold-change 1.22), RORA (fold-change 1.47), SLC16A1 (MTO1) (fold-change 5.25); RPL36 (fold-change 2.11), SH2B3 (fold-change 2.28), TNFRSF10B (fold-change 2.0); and TNFRSF12A (fold-change 1.54) (*n* = 535; *p* < 0.001) (Figure 7b–h). Even though several ZNF433 target genes were associated with tumorigenesis, gene pathway analysis using the PANTHER web tool revealed no statistically significant pathways enriched within the ZNF433 target gene list. Nonetheless, the largest group of genes were involved in the cellular process (GO:009987) (Figure 7h), and the largest group within the cellular process category was genes involved in cellular metabolic processes (GO: 0044237) (Figure 7i).

### 3.7. Pan-Cancer Analysis of ZNF433 Target Gene Expression

We examined ZNF433 expression across cancer types (Table 3). ZNF433 was significantly downregulated (*p* < 0.05) in the head and neck, clear-cell renal cell carcinoma, renal papillary, renal chromophobe, lung, and thyroid cancers. Conversely, ZNF433 was significantly upregulated (*p* < 0.05) in bladder, cholangiocarcinoma, liver, and uterine cancers.

Abbreviations used include: ACC, adrenocortical carcinoma; BLCA, bladder urothelial carcinoma; BRCA, breast invasive carcinoma; CESC, cervical squamous cell carcinoma and endocervical adenocarcinoma; CHOL, cholangiocarcinoma; COAD, colon adenocarcinoma; ESCA, esophageal carcinoma; GBM, glioblastoma multiforme; HNSC, head and neck squamous cell carcinoma; KICH, kidney chromophobe; KIRC, kidney renal clear cell carcinoma; KIRP, kidney renal papillary cell carcinoma; LIHC, liver hepatocellular carcinoma; LUAD, lung adenocarcinoma; LUSC, lung squamous cell carcinoma; PAAD, pancreatic adenocarcinoma; PCPG, pheochromocytoma and paraganglioma; PRAD, prostate adenocarcinoma; READ, rectum adenocarcinoma; SKCM, skin cutaneous melanoma; STAD, stomach adenocarcinoma; THCA, thyroid carcinoma; UCEC, uterine corpus endometrial carcinoma.

## 4. Discussion

Though highly treatable if the diagnosis is early, clear-cell renal cell carcinoma (ccRCC) is the most common and aggressive form of urological cancers [31]. Unfortunately, 20–30% of renal carcinomas are detected in later stages and are often associated with treatment challenges and poorer prognosis. Thus, there is a need to identify prognostic markers which can be used to detect ccRCC. Zinc-finger proteins are the largest family of transcription factors. Moreover, given that cancers are characterized by abnormal gene transcription, these proteins represent a significant opportunity to identify molecular signatures, which could serve as either prognostic or predictive factors for neoplastic diseases, including clear-cell renal cell carcinoma. Indeed, Krüppel-associated box domain zinc-finger proteins (KRAB-ZFPs) are often dysregulated in cancer cells [8]. For instance, uterine corpus endometrial carcinoma, colon and rectal adenocarcinomas, and skin cutaneous melanoma possess significant mutations in the Krüppel-associated box (KRAB) repressor domains in the Cys2His2 subfamily of zinc-finger proteins [32]. KRAB-ZNFs such as ZNF695, ZNF468, ZNF714, ZNF320, ZNF273, ZNF525, ZNF530, ZNF643, ZNF138, ZNF92, ZNF200, ZNF707, ZNF205, ZNF485, ZNF354A, and ZNF789 are differentially expressed across multiple cancer cohorts [8,11]. Moreover, recent studies suggest that KRAB-ZNFs can modulate tumorigenesis. For example, ZNF217 overexpression is associated with poorly differentiated tumors and the progression of ovarian cancers [33]. Restoration of KRAB-ZNF382 expression in silenced ESCC cells suppresses tumor-cell proliferation and metastasis by inducing cell apoptosis [34]. Overexpression of ZNF671 inhibited EMT, migration, and invasion of CNS cancers, lung cancer, melanoma, and breast carcinoma in vitro [18]. Taken collectively, KRAB-ZNF proteins play a significant role in tumorigenesis, and altered expression may serve as biomarkers, oncogenes, or tumor-suppressor genes.

In this study, data indicated that ZNF433 behaved in a consistent manner, with it being a prognostic marker and/or a putative tumor-suppressor gene. ZNF433 belongs to the Krüppel-associated box (KRAB) C2H2-type zinc-finger protein subfamily, and like many other members of this subfamily, is part of a cluster of KRAB-ZNFs found on chromosome 19q13 [35]. Because of the sheer number of ZNF genes located in this region, the functions of these proteins and their roles in tumorigenesis have not been fully elucidated. ZNF433 gene and protein expression are reduced in clear-cell renal cell carcinoma (Figure 1a,b). Expression is stage- and grade-specific, with mRNA levels being significantly suppressed in higher stages (III and IV) and grades (III and IV) compared to lower grades and stages (I and II) (Figure 2a–d). These data strongly suggest that ZNF433 may mediate tumorigenesis, and loss of function is associated with cancer progression and aggressiveness. Indeed, transcript expression decreased progressively with metastatic nodal involvement (Figure 2c) and was significantly lower (Figure 2d) in the more aggressive, higher-risk ccB renal carcinoma subtype [36]. In addition, lower transcription expression was associated with low patient overall survival (Figure 3a), thereby suggesting that ZNF433 may play a protective role against tumorigenesis in normal kidney tissues.

Since ZNF433 levels are significantly reduced in ccRCC, we investigated whether gene suppression could be due to epigenetic mechanisms. The ZNF433 promoter and CpG foci were found to be significantly hypermethylated. Moreover, hypermethylation was negatively correlated with mRNA expression, thereby suggesting that ZNF433 suppression may be due, in part, to aberrant methylation. Because ccRCC is also characterized by mutations in genes that are involved in chromatin remodeling and epigenetic regulations such as BAP1, KDM5C, MTOR, PBRM1, SETD2, and VHL [37,38,39,40,41,42], we examined the effects of the most commonly altered genes on ZNF433 expression. ZNF433 transcripts were significantly reduced in BAP1, SETD2, and KDM5C mutants, with BAP1 mutants exerting the greatest suppressive effects. The BAP1 gene encodes a ubiquitin carboxyl-terminal hydrolase, a deubiquitinase enzyme that regulates a number of processes through modification of histones and regulating chromatin scaffolding. BAP1 functions as a major tumor suppressor that mediates DNA damage repair, cell-cycle control, chromatin modification, programmed cell death, and the immune response [43]. Indeed, loss function in the BAP1 gene is associated with several aggressive cancers, including clear-cell renal cell carcinomas [43]. SETD2 encodes a methyltransferase that specifically trimethylates lysine-36 of histone H3 (H3K36me3), whereas KDM5c (also known as JARID1C) is a gene that encodes an H3K4me2/3 demethylase that plays a central role in transcriptional repression and can mediate cancer progression [44]. These three genes are all involved in epigenetic controls and chromatin modeling, suggesting that histone modification, like DNA methylation, is essential to transcriptional control of ZNF433.

Given that ZNF433 is a putative transcription factor, we identified potential target genes using the GSEA Molecular Signature Database (Table 4). We examined the expression of ZNF433 target genes in clear-cell renal cell carcinoma. Several target genes were differentially expressed, and associated with cancer suppression, progression, or tumorigenesis (Figure 6a,b). The most notable target genes (Figure 7b–h) were potential oncogenes/promoters of tumorigenesis (COPS3, RORA, SLC16A1), and potential tumor suppressors (RPL36, SH2B3, TNFRSF10B, TNFRSF12A) [45,46,47,48]. In addition, we used PANTHER to annotate ZNF433 target genes (Figure 7h,i). The largest portion of genes (29.7%) were associated with biological processes (GO:0009987) (Figure 7i), and within the category of biological processes, the largest number of genes were involved in cellular metabolic processes (GO:0044237) (Figure 7h,i). However, given the small numbers of possible target genes, no pathway or process was statistically over-represented. Nonetheless, it should be noted that clear-cell renal cell carcinoma is especially characterized by impaired cellular metabolism and metabolic reprogramming characterized by increased glycolysis and lactate production, and abnormal accumulations of lipids and reduced oxidative phosphorylation [49,50].

ZNF433 may be involved in cellular metabolism. Indeed, pathway analysis of the KEGG database suggested that genes positively co-expressed with ZNF433 are associated with metabolism. Though not rising to the level of significance as defined by *p*-values and FDR less than 0.05, positively correlated genes in the GO Biological Processes database were mainly associated with cellular energetics and mitochondrial function, further suggesting that ZNF433 may be involved in the regulation of cellular energetics. It should be noted that altered cellular energetics may impact the ccRCC tumor microenvironment by regulating angiogenesis and inflammatory signatures [51]. Clear-cell renal cell carcinoma is highly infiltrated with T cells, and therapeutic interventions such as the use of MTOR inhibitors and checkpoint inhibitors (PD-1 and PD-L1) have been shown to be effective in the management of ccRCC [52]. However, recent studies have shown that T cells may become dysfunctional or “exhausted” due to aberrant mitochondrial functions and cellular energetics [52,53,54,55]. Interestingly, GSEA analysis of the Gene Ontology Biological Processes database, as well as the KEGG database, revealed that pathways that were enriched in immune function were over-represented in genes negatively co-expressed with ZNF433. In the case of GO Biological Processes, all enriched pathways associated with inverse expression of ZNF433 were related to immune function. Hence, ZNF433 may be intimately involved in the dysregulation of cellular energetic and immune function.

Lastly, we examined ZNF433 expression across multiple cancers to ascertain if ZNF433 may be a common tumor marker (Table 3). ZNF433 expression was upregulated (*p* < 0.05) in bladder urothelial carcinoma, cholangiocarcinoma, glioblastoma multiforme, liver hepatocellular carcinoma, pheochromocytoma and paraganglioma, prostate adenocarcinoma, and uterine corpus endometrial carcinoma; while expression was suppressed (*p* < 0.05) in head and neck squamous cell carcinoma, kidney chromophobe, kidney renal clear-cell carcinoma, kidney renal papillary cell carcinoma, lung adenocarcinoma, lung squamous cell carcinoma, and thyroid carcinoma, thereby indicating that ZNF433 expression is altered across multiple tumor cohorts and thus may play a general role in cancer formation and progression.

## 5. Conclusions

In conclusion, ZNF433 is a member of the KRAB-ZFTF subfamily, which is differentially expressed in several cancers; however, poor prognosis is largely associated with low expression in kidney cancers. Although its role in renal carcinoma is yet to be fully defined, it is clear that this gene suppression is associated with higher cancer stages, tumor grades, degree of metastasis, and more aggressive subtypes of ccRCC. Therefore, ZNF433 represents a predictive risk factor for tumor progression, and may prove to be a possible therapeutic target in the treatment of ccRCC. Though additional large-scale validation is warranted, we anticipate that assessing ZNF433 mRNA expression in biopsies will be most beneficial as a diagnostic tool for screening and identifying patients at higher risks of developing the aggressive ccRCC subtypes. Given the scarcity of information regarding the transcriptional targets of ZNF433, comprehensive analysis is required to identify and validate ZNF433 gene targets, as well as transcription regulators of ZNF433, particularly in clear-cell renal cell carcinomas.

## Figures and Tables

**Table 1:** Clinicopathological features of the ccRCC TCGA dataset. A *t*-test was conducted to analyze differential gene expression of ZNF433 between genders. ANOVA was used to analyze ZNF433 gene expression across clinicopathological features. Statistical significance was set at *p* < 0.05. N.S. = not significant (*p* > 0.05).

Clinicopathological Features	N	Statistics
Gender		N.S.
Male	385	
Female	188	
Histological Grade		*p* < 0.001
Normal	72	
1	14	
2	229	
3	206	
4	76	
Pathological Stage		*p* < 0.001
Normal	72	
1	267	
2	57	
3	123	
4	84	
Metastasis		*p* < 0.001
Normal	72	
N0	240	
N1	16	
Age		N.S.
21–40 years	26	
41–60 years	238	
61–80 years	246	
81+ years	23	

**Table 2:** Univariate and multivariant Cox regression analysis of age, gender, pathological stage, histological grade in relation to overall survival in ccRCC patients. The statistical significance was set at *p* < 0.05.

Clinicopathological Feature	Univariate Analysis HR (95% CI) *p*-Value	Multivariate Analysis HR (95% CI) *p*-Value
ZNF433	0.6227 (0.4593–0.8440)	0.7390 (0.6987–1.3065)
	*p* = 0.0023	*p* = 0.0597
Gender	0.9508 (0.7003–1.2910)	0.9711 (0.7094–1.3292)
	*p* = 0.7466	*p* = 0.8547
Age	1.7589 (1.2979–2.3835)	1.5889 (1.1612–2.1742)
	*p* = 0.0003	*p* = 0.0038
Stage	3.8179 (2.7869–5.2303)	3.3766 (2.4441–4.6649)
	*p* < 0.0001	*p* < 0.001
Grade	1.5172 (1.1152–2.0639)	1.2429 (0.9088–1.6697)
	*p* = 0.0079	*p* = 0.1734

**Table 3:** Expression profile of ZNF433 in different cancers. Statistical significance was set at *p* < 0.05.

Tumor	Normal	Change	Significance (*p*-Value)
BLCA.Tumor (*n* = 408)	BLCA.Normal (*n* = 19)	Upregulation	0.016096887
BRCA.Tumor (*n* = 1093)	BRCA.Normal (*n* = 112)	Downregulation	0.446186328
CESC.Tumor (*n* = 304)	CESC.Normal (*n* = 3)	Downregulation	0.210693518
CHOL.Tumor (*n* = 36)	CHOL.Normal (*n* = 9)	Upregulation	0.005768157
COAD.Tumor (*n* = 457)	COAD.Normal (*n* = 41)	Upregulation	0.792680029
ESCA.Tumor (*n* = 184)	ESCA.Normal (*n* = 11)	Downregulation	0.164913994
GBM.Tumor (*n* = 153)	GBM.Normal (*n* = 5)	Upregulation	0.001122476
HNSC.Tumor (*n* = 520)	HNSC.Normal (*n* = 44)	Downregulation	6.12 × 10^−8^
KICH.Tumor (*n* = 66)	KICH.Normal (*n* = 25)	Downregulation	8.04 × 10^−5^
KIRC.Tumor (*n* = 533)	KIRC.Normal (*n* = 72)	Downregulation	1.13 × 10^−23^
KIRP.Tumor (*n* = 290)	KIRP.Normal (*n* = 32)	Downregulation	2.86 × 10^−9^
LIHC.Tumor (*n* = 371)	LIHC.Normal (*n* = 50)	Upregulation	7.93 × 10^−11^
LUAD.Tumor (*n* = 515)	LUAD.Normal (*n* = 59)	Downregulation	0.039291777
LUSC.Tumor (*n* = 501)	LUSC.Normal (*n* = 51)	Downregulation	1.16 × 10^−5^
PAAD.Tumor (*n* = 178)	PAAD.Normal (*n* = 4)	Downregulation	0.112226334
PCPG.Tumor (*n* = 179)	PCPG.Normal (*n* = 3)	Upregulation	0.032947137
PRAD.Tumor (*n* = 497)	PRAD.Normal (*n* = 52)	Upregulation	0.028391067
READ.Tumor (*n* = 166)	READ.Normal (*n* = 10)	Downregulation	0.101167394
SKCM.Tumor (*n* = 103)	SKCM.Metast (*n* = 368)	Downregulation	0.826588455
STAD.Tumor (*n* = 415)	STAD.Normal (*n* = 35)	Downregulation	0.433244729
THCA.Tumor (*n* = 501)	THCA.Normal (*n* = 59)	Downregulation	1.98 × 10^−11^
UCEC.Tumor (*n* = 545)	UCEC.Normal (*n* = 35)	Upregulation	0.015728415

**Table 4:** List of predicted ZNF433 target genes identified using the GSEA Molecular Signature Database. These are genes containing one or more binding sites for UniProt: Q8N7K0 (ZNF433) in their promoter regions (TSS −1000, +100 bp) as identified by GTRD version 20.06 ChIP-seq harmonization.

Gene Symbol	Official Name
ACER3	alkaline ceramidase 3
ALDOA	aldolase, fructose-bisphosphate A
ANGPTL6	angiopoietin-like 6
AP1S3	adaptor related protein complex 1 subunit sigma 3
ASS1P5	argininosuccinate synthetase 1 pseudogene 5
AURKB	aurora kinase B
BORCS6	BLOC-1 related complex subunit 6
C6orf141	chromosome 6 open reading frame 141
CCDC65	coiled-coil domain containing 65
COPS3	COP9 signalosome subunit 3
COQ10A	coenzyme Q10A
CROCCP3	CROCC pseudogene 3
CWC25	CWC25 spliceosome associated protein homolog
CYP1B1-AS1	CYP1B1 antisense RNA 1
DAGLB	diacylglycerol lipase beta
DHTKD1	dehydrogenase E1 and transketolase domain containing 1
EXOSC2	exosome component 2
FABP5P3	fatty-acid-binding protein 5 pseudogene 3
FCHO2	FCH and mu domain-containing endocytic adaptor 2
FES	FES proto-oncogene, tyrosine kinase
FMN2	formin 2
FRMD7	FERM domain containing 7
GALNTL5	polypeptide N-acetylgalactosaminyltransferase like 5
GIT2	GIT ArfGAP 2
GLG1	Golgi glycoprotein 1
GPR1-AS	GPR1 antisense RNA
GXYLT2	glucoside xylosyltransferase 2
HAPLN2	hyaluronan and proteoglycan link protein 2
HEBP2	heme binding protein 2
KAT8	lysine acetyltransferase 8
LINC01235	long intergenic non-protein coding RNA 1235
LINC01641	long intergenic non-protein coding RNA 1641
LUZP1	leucine zipper protein 1
MED21	mediator complex subunit 21
MTFMT	mitochondrial methionyl-tRNA formyl transferase
MTND1P14	MT-ND1 pseudogene 14
MTO1	mitochondrial tRNA translation optimization 1
NLE1	notchless homolog 1
NOL6	nucleolar protein 6
NUCB1-AS1	NUCB1 antisense RNA 1
OR1X5P	olfactory receptor family 1 subfamily X member 5 pseudogene
QSER1	glutamine and serine rich 1
RN7SL93P	RNA, 7SL, cytoplasmic 93, pseudogene
RND1	Rho family GTPase 1
RNU6-1003P	RNA, U6 small nuclear 1003, pseudogene
RNU6-166P	RNA, U6 small nuclear 166, pseudogene
RORA	RAR related orphan receptor A
RPL32P27	ribosomal protein L32 pseudogene 27
RPL36	ribosomal protein L36
RRN3P1	RRN3 pseudogene 1
SDC4	syndecan 4
SH2B3	SH2B adaptor protein 3
SLC6A1	solute carrier family 6-member 1
SNHG30	small nucleolar RNA host gene 30
SPATS2L	spermatogenesis associated serine rich 2 like
STX4	syntaxin 4
SYCE2	synaptonemal complex central element protein 2
TMEM98	transmembrane protein 98
TNFRSF10B	TNF receptor superfamily member 10b
TNFRSF12A	TNF receptor superfamily member 12A
TRAV15	T cell receptor alpha variable 15 (pseudogene)
TRIM15	tripartite motif containing 15
TTC1	tetratricopeptide repeat domain 1
TTLL13P	tubulin tyrosine ligase like 13, pseudogene
WNT8A	Wnt family member 8A
ZNF675	zinc-finger protein 675

## Data Availability

Datasets utilized to analyze the clinicopathological features of ZNF433 and target genes can be assessed with the UALCAN webtool (http://ualcan.path.uab.edu/analysis.html, accessed on 5 May 2021)**.** Data collection for the UALCAN analysis webtool has been previously described in detail [18]. Specifically, for clear-cell renal cell carcinoma, the KIRC-TCGA was downloaded from the TCGA database. To investigate ZNF433 and its putative target associated with mRNA expression, tumor stage, grade, and subtype, 533 tumor samples and 72 normal tissues were taken for analysis. Due to the lack of cancer stage information, two samples were excluded, leaving 531 tumor samples for further analysis. For analysis of gene expression relative to tumor grade, eight samples were excluded due to inefficient information regarding histological grade, leaving 525 tumor samples. For ZNF433 expression in metastatic tissues, 277 samples were excluded because cancer in nearby lymph nodes (Nx) could not be measured, leaving 256 tumor samples for analysis. Lastly, 153 samples were excluded for analysis of ccRCC subtypes because no information regarding subtypes was available, leaving 380 tumor samples.
